# Associations of heavy metal exposure with diabetic retinopathy in the U.S. diabetic population: a cross-sectional study

**DOI:** 10.3389/fpubh.2024.1401034

**Published:** 2024-08-01

**Authors:** Chunren Meng, Chufeng Gu, Chunyang Cai, Shuai He, Dongwei Lai, Qinghua Qiu

**Affiliations:** ^1^Department of Ophthalmology, Tong Ren Hospital, Shanghai Jiao Tong University School of Medicine, Shanghai, China; ^2^Department of Ophthalmology, Shanghai General Hospital, Shanghai Jiao Tong University School of Medicine, National Clinical Research Center for Eye Diseases; Shanghai Clinical Research Center for Eye Diseases, Shanghai, China; ^3^Department of Ophthalmology, Fuzhou University Affiliated Provincial Hospital, Fuzhou, Fujian, China; ^4^Department of Ophthalmology, Shigatse People’s Hospital, Shigatse, Xizang, China; ^5^High Altitude Ocular Disease Research Center of Shigatse People’s Hospital and Tongren Hospital Affiliated to Shanghai Jiao Tong University School of Medicine, Shanghai, China

**Keywords:** heavy metal exposure, diabetic retinopathy, public health, NHANES, risk factors

## Abstract

**Background:**

Mounting evidence suggests a correlation between heavy metals exposure and diabetes. Diabetic retinopathy (DR) is a prevalent and irreversible complication of diabetes that can result in blindness. However, studies focusing on the effects of exposure to heavy metals on DR remain scarce. Thus, this study aimed to investigate the potential correlation between heavy metals exposure and DR.

**Methods:**

A total of 1,146 diabetics from the National Health and Nutrition Examination Survey (NHANES) between 2005 and 2018 were included in this study. Heavy metal levels were measured via urine testing. Weighted logistic regression, Bayesian kernel machine regression (BKMR), weighted quantile sum (WQS) regression, and restricted cubic spline (RCS) were utilized to investigate the potential relationships between exposure to 10 heavy metals and DR. Finally, subgroup analysis was conducted based on the glycemic control status.

**Results:**

Among the 1,146 participants, 239 (20.86%) were diagnosed with DR. Those with DR had worse glycemic control and a higher prevalence of chronic kidney disease compared to those without DR. Moreover, both the WQS regression and BKMR models demonstrated a positive relationship between exposure to mixed heavy metals and the risk of DR. The results of weighted logistic regression revealed a positive correlation between cobalt (Co) and antimony (Sb) exposure and the risk of DR (OR = 1.489, 95%CI: 1.064–2.082, *p* = 0.021; OR = 1.475, 95% CI: 1.084–2.008, *p* = 0.014), while mercury (Hg) exposure was found to promote DR exclusively in the group with good glycemic control (OR = 1.509, 95% CI: 1.157–1.967, *p* = 0.003). These findings were corroborated by the results of the RCS analysis.

**Conclusion:**

Heavy metal exposure is associated with an increased risk of DR, especially Sb, Co, and Hg exposure. Nevertheless, well-designed prospective studies are warranted to validate these findings.

## 1 Introduction

Diabetic retinopathy (DR) is a prevalent microvascular complication of diabetes mellitus that affects approximately one-third of diabetic patients (1). It causes varying degrees of visual impairment (2), which significantly impacts the quality of life of patients and imposes substantial economic burdens on society (3). Notably, its pathogenesis is complex and multifaceted, including oxidative stress, inflammation, and mitochondrial disorders, among others (4). At present, there is a pressing need to identify the risk factors and intervention strategies for DR in order to enhance the prognosis of patients with DR.

As is well documented, heavy metals are ubiquitously present in the air, soil, water, food, and manufactured products (5–9). Exposure to heavy metals may increase the risk of various ocular diseases, including DR, age-related macular degeneration (AMD), glaucoma, and cataracts (10–13). Zhu et al. demonstrated that the accumulation of serum cesium (Cs) and cadmium (Cd) was significantly correlated with the risk of developing DR (10). Similarly, the findings of Li et al. indicated that exposure to certain heavy metals, including lithium (Li), Cd, strontium (Sr), and magnesium (Mg), may increase the risk of developing proliferative DR, whereas selenium (Se) appears to be a protective factor (14). Zhang et al. observed a significant negative correlation between serum manganese (Mn) levels and DR prevalence in individuals with type 2 diabetes mellitus in the United States (15). However, the correlation between serum Cd, mercury (Hg), and lead (Pb), and DR was not statistically significant (15). Other studies have determined a potential association between cobalt (Co), barium (Ba), molybdenum (Mo), antimony (Sb), thallium (Tl), and tungsten (Tu) and the risk of diabetes (16–18), but their relationship with DR remains elusive. Although previous studies have preliminarily explored the link between heavy metals and DR, certain limitations remain. For instance, earlier studies exclusively investigated the association between the levels of serum heavy metals and DR risk, with a lack of research on the effect of urinary heavy metals on DR. Serum heavy metal levels may correlate with recent exposure, whereas urine heavy metal concentrations reflect long-term exposure (19). Furthermore, heavy metals are frequently co-exposed in the environment, and interactions between metals may also have an impact on human health (20, 21). However, studies on co-exposure to heavy metals and DR risk are lacking. Additionally, there is a lack of epidemiological studies to elucidate the effects of other heavy metals, such as Co and Sb, on the risk of developing DR.

The present study extracted U.S. demographic data from the National Health and Nutrition Examination Survey (NHANES) between 2005 and 2018 to investigate the relationship between heavy metals and the risk of DR. A total of 10 urinary heavy metals, namely Ba, Cd, Co, Cs, Mo, Pb, Sb, Tl, Tu, and Hg were analyzed. The effect of single and multiple metals on DR risk was evaluated using weighted logistic regression analysis. Furthermore, weighted quantile sum (WQS) regression and Bayesian kernel machine regression (BKMR) model were applied to investigate the relationship between heavy metals co-exposure and DR. In addition, dose–response relationships between heavy metals and DR were explored using restricted cubic spline (RCS) regression. Lastly, subgroup analysis was conducted based on glycemic control levels. Our findings are anticipated to provide new epidemiological evidence to enhance the understanding of the correlation between heavy metals and DR and assist in the prevention of DR.

## 2 Materials and methods

### 2.1 Study design

#### 2.1.1 Participants

**Figure 1:**
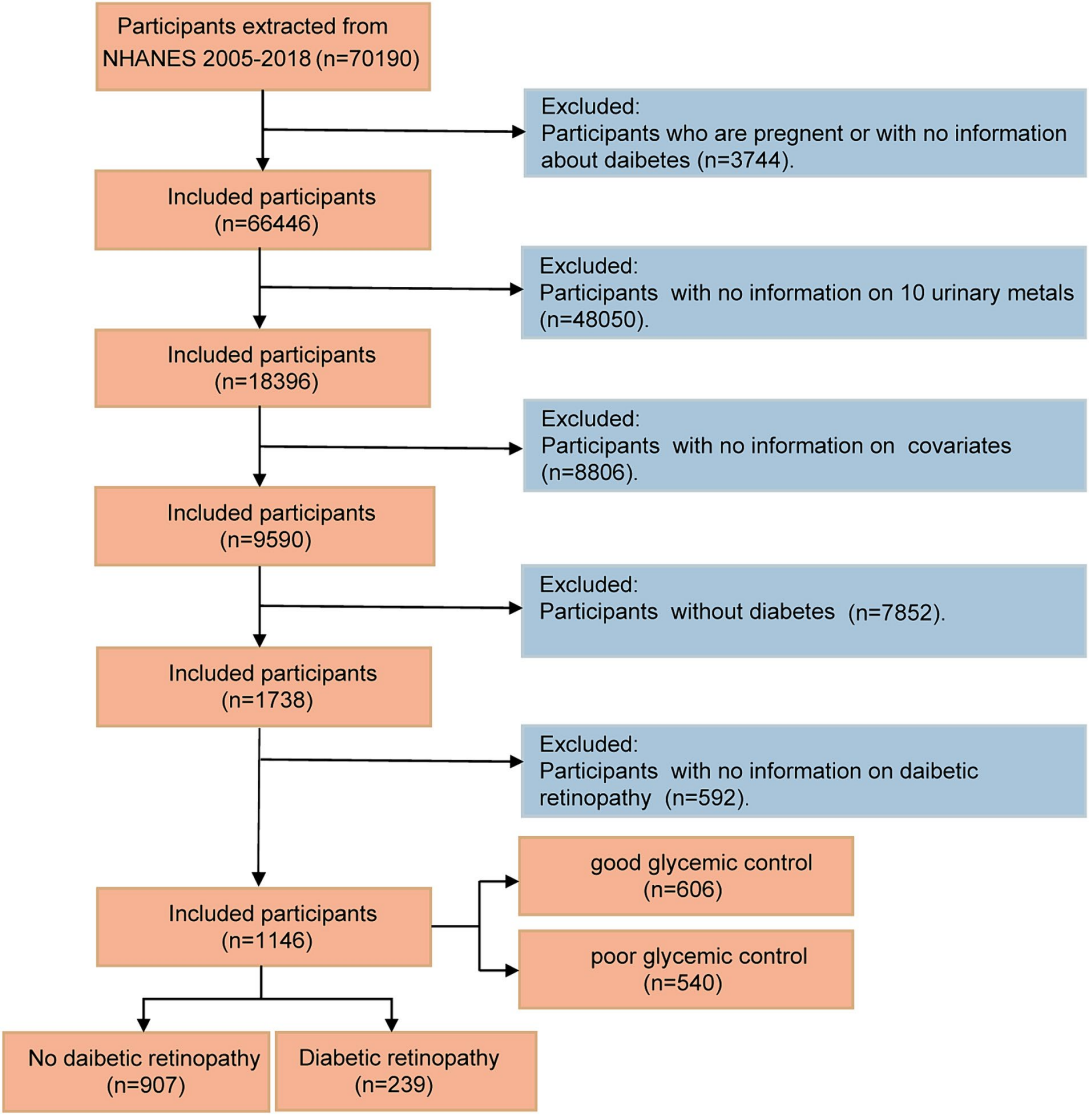
Flow diagram of the screening and enrollment of study participants.

The NHANES aimed to assess the health and nutritional status of the US population. By employing a complex multistage probability sampling technique, the NHANES collects information on the nation’s civilian population every 2 years (22). In the current study, data derived from NHANES between 2005 and 2018 (seven NHANES cycles) were analyzed, given that participants underwent relatively comprehensive urine testing for heavy metals during these cycles. NHANES was approved by the Ethics Review Committee at the National Center for Health Statistics, and all participants provided informed consent. Among the 70,190 participants across the NHANES cycles conducted between 2005 and 2018, several groups were excluded according to the following criteria: (1) participants who were pregnant or lacked data on diabetes (*n* = 3,744); (2) participants with incomplete urinary metal levels (*n* = 48,050); (3) participants who had missing covariate data (*n* = 8,806); (4) non-diabetic individuals (*n* = 7,852); and (5) participants with other missing information on diabetic retinopathy (*n* = 592). The final study cohort comprised 1,146 subjects, as illustrated in Figure 1.

#### 2.1.2 Definitions of diabetes and DR

Diagnostic criteria for diabetes comprised any of the following: (1) diagnosis by medical professionals during a non-gestational period, (2) hemoglobin A1c level (HbA1c) (%) ≥ 6.5, (3) fasting plasma glucose level (FPG) ≥ 7.0 mmol/L, (4) random blood glucose level ≥ 11.1 mmol/L, (5) 2-h oral glucose tolerance test (OGTT) blood glucose ≥11.1 mmol/L, and (6) receiving anti-diabetic medication (23). DR was determined through self-report using a dichotomous approach. Participants were informed by medical professionals that diabetes had affected their eyes (24).

### 2.2 Metal measurement

Between 2005 and 2018, the levels of 10 heavy metals, namely Ba, Cd, Co, Cs, Mo, Pb, Sb, Tl, Tu, and Hg, were detected in urine samples. The NHANES website provides all laboratory methods and quality control information. Briefly, the concentration of 10 urinary metals was determined using inductively coupled plasma-mass spectrometry (ICP-MS). If the metal concentration was below the limit of detection (LOD), the LOD divided by the square root of two was used as the surrogate. In addition, all urinary metal levels were normalized to urinary creatinine and reported as μg/g creatinine (25).

### 2.3 Covariates

Demographic characteristics [gender, age, ethnicity, educational background, and family poverty income ratio (PIR)], along with data on body mass index (BMI), HbA1c levels, history of hypertension and chronic kidney disease (CKD), smoking status, and drinking habits, were acquired through either home interviews or laboratory assessments. Ethnicity was classified into five groups: non-Hispanic white, non-Hispanic black, Mexican American, other Hispanic, and other race/multiracial. PIR was categorized into three levels: <1.30, 1.30–3.5, and ≥ 3.5 (21). Similarly, BMI was divided into three levels: <25, 25–30, and > 30 kg/m^2^. Glycemic control was classified as well (HbA1c < 7%) and poor (HbA1c ≥7%). Drinking status was self-reported by the participants. Smoking status was determined through the evaluation of serum cotinine levels, with a cutoff value of ≤0.011 ng/mL for nonsmokers and higher levels indicating smoking status for both active and second-hand smokers (26). Hypertension was defined as any of the following: self-reported hypertension, ever or currently taking anti-hypertensive drugs, a systolic blood pressure over 140 mmHg, or a diastolic blood pressure exceeding 90 mmHg. CKD was defined as any of the following: an estimated glomerular filtration rate (eGFR) < 60 mL/min/1.73 m^2^ or the presence of elevated albuminuria (urine albumin creatinine ratio ≥ 30 mg/g) (27).

### 2.4 Statistical analysis

WTSA2YR is considered the appropriate NHANES sampling weight to analyze data on urinary heavy metals. Given the complex sampling design of NHANES, weights (1/7 * WTSA2YR) were constructed in accordance with the analytic guidelines of NHANES. Weighted means (standard errors) were employed to present continuous variables, while unweighted frequencies (weighted percentages) were utilized to present categorical variables. Baseline comparisons were made based on DR status stratification. The *t*-test was used to compare continuous variables, whereas the chi-square test was used to compare categorical variables. Given the upward trend in heavy metal concentration in the human body, an Ln transformation was performed on heavy metal concentration data to approximate a normal distribution (continuous variable) and divided the heavy metal concentration data into quartiles. The relationships between the concentrations of the 10 metals were determined using Pearson correlation analysis.

First and foremost, weighted logistic regression was employed to explore the impact of each metal on the risk of DR. The reference group was set as the first quartile (Q1), and the results were expressed as odds ratios (ORs) and their corresponding 95% confidence intervals (CIs). All covariates, including age, gender, ethnicity, educational background, PIR, glycemic control, smoking and drinking status, BMI, hypertension, and CKD, were adjusted. Furthermore, a weighted logistic regression analysis encompassing all heavy metals was conducted to adjust for the effects of other metals.

Secondly, to assess the combined effect of exposure to multiple metals on DR risk, a WQS regression analysis was carried out. This method was selected owing to its effective characterization of environmental mixtures (28). The R package (“gWQS”) was utilized to compute the WQS index, which is a weighted sum of the concentrations of individual heavy metals (21). The WQS index (ranging from 0 to 1) indicated the level of mixed exposure to the 10 heavy metals. The weight of each metal reflected its relative importance for the risk of DR. The WQS analysis results provided information about the concurrent influence of adding a quartile to heavy metals mixtures on DR risk.

BKMR is a developing statistical method that utilizes kernel functions to effectively model the individual and joint impacts of mixture exposure on health results (29). The common influences of heavy metal mixtures on DR were examined by analyzing the DR estimates for every 5 percent increase/decrease in the median concentration of metal mixtures (reference) (25). The posterior probability of inclusion (PIP) was calculated with a threshold of 0.5 to assess the relative contribution of each metal component to the outcome (30). The BKMR model was generated via the R package “bkmr” through 10,000 iterations (31).

Subsequently, an RCS regression analysis was conducted using the R package “rms” to investigate the dose–response association of heavy metal exposure with DR risk. RCS regression was used to analyze both the linear and nonlinear relationships between heavy metals levels and DR risk (32). The number of nodes was selected to maintain the best fit and prevent overfitting the principal spline, with a range of 3–7 nodes considered according to the minimum absolute value of Akaike’s information criterion (33). Finally, the 3 knots corresponding to the 10th, 50th, and 90th percentiles were chosen.

Finally, the same statistical analysis procedures previously outlined were applied to the subgroups based on glycemic control (well-controlled group: HbA1c value <7%, poorly-controlled group: HbA1c value ≥7%).

Statistical analyses were performed using R software (v4.3.1), with *p*-values less than 0.05 considered statistically significant.

## 3 Results

### 3.1 Study population characteristics

This study included 1,146 participants from seven NHANES cycles, comprising 550 women (weighted survey sample of 7,898,021) and 596 men (weighted survey sample of 8,162,870). Among them, 239 (20.86%) were diagnosed with DR, including 134 males and 105 females. Table 1 presents a summary of the baseline characteristics of the study participants with and without DR. Consistent with the findings of previously published studies, our study confirmed that participants with DR exhibited poorer glycemic control than those with diabetes without DR. Furthermore, the prevalence of CKD was higher in individuals with DR compared to those without DR. The two groups were comparable in age, gender, ethnicity, educational background, PIR, BMI, drinking and smoking status, and prevalence of hypertension.

**Table 1:** Baseline characteristics of study population by DR status.

Variable	Total (*N* = 1,146)	Non-DR (*N* = 907)	DR (*N* = 239)	*p* value
Age, years	58.514 (0.549)	58.745 (0.606)	57.578 (1.183)	0.378
Sex, *n* (%)				0.342
Female	550 (49.175)	445 (50.328)	105 (44.502)	
Male	596 (50.825)	462 (49.672)	134 (55.498)	
Ethnicity, *n* (%)				0.473
Mexican American	217 (9.219)	173 (9.275)	44 (8.991)	
Non-Hispanic Black	275 (13.323)	221 (13.453)	54 (12.796)	
Non-Hispanic White	425 (64.961)	344 (65.232)	81 (63.861)	
Other Hispanic	120 (5.651)	91 (5.847)	29 (4.855)	
Other race	109 (6.846)	78 (6.192)	31 (9.496)	
Education, *n* (%)				0.79
Greater than high school	478 (49.391)	378 (49.696)	100 (48.154)	
High school or below	668 (50.609)	529 (50.304)	139 (51.846)	
BMI (kg/m^2^), *n* (%)				0.82
<25	141 (10.047)	103 (9.666)	38 (11.590)	
25–30	320 (26.132)	253 (26.098)	67 (26.271)	
≥30	685 (63.821)	551 (64.236)	134 (62.139)	
PIR, *n* (%)				0.481
≤1.30	408 (24.080)	310 (23.297)	98 (27.255)	
1.30–3.50	473 (41.302)	386 (42.522)	87 (36.355)	
>3.50	265 (34.619)	211 (34.181)	54 (36.390)	
Drinking, *n* (%)				0.088
Never	204 (15.090)	149 (13.444)	55 (21.764)	
Former	302 (22.077)	235 (22.170)	67 (21.699)	
Now	640 (62.833)	523 (64.386)	117 (56.537)	
Smoking, *n* (%)				0.833
Non-smoker	314 (30.365)	247 (30.624)	67 (29.313)	
Smoker	832 (69.635)	660 (69.376)	172 (70.687)	
Glycemic control, *n* (%)				**<0.0001**
Well-controlled	606 (56.183)	511 (60.711)	95 (37.829)	
Poorly controlled	540 (43.817)	396 (39.289)	144 (62.171)	
Hypertension, *n* (%)				0.346
No	301 (27.458)	242 (28.240)	59 (24.287)	
Yes	845 (72.542)	665 (71.760)	180 (75.713)	
CKD, *n* (%)				**0.002**
No	681 (64.623)	574 (67.894)	107 (51.365)	
Yes	465 (35.377)	333 (32.106)	132 (48.635)	

Continuous variables were presented as weighted means (standard errors) and categorical variables are expressed as unweighted numbers (weighted percentages). DR, diabetic retinopathy; BMI, body mass index; N, numbers of subject; %, weighted percentage; NHANES, National Health and Nutrition Examination Survey; PIR, Poverty Income Ratio; CKD, chronic kidney disease. *P* value was calculated by chi-squared test and Student’s t-test. Bold: *p* < 0.05.

### 3.2 Distributions and correlations of the 10 heavy metals

Supplementary Table 1 lists the distribution of concentrations for 10 heavy metals, with detection rates exceeding 93.0% for each metal. Interestingly, Mo was the most metal with the highest level. Additionally, patients with DR had significantly higher levels of Sb compared to those without DR (*p* = 0.021). The correlations between the 10 heavy metals are detailed in Supplementary Figure 1. Co and Tl (*r* = 0.58), Co and Ba (*r* = 0.43), Cs and Ba (*r* = 0.35), Co and Cs (*r* = 0.34), Tu and Mo (*r* = 0.34), and Tl and Ba (*r* = 0.31) exhibited positive correlations. Other metals had relatively weak correlations.

### 3.3 Association of heavy metals with DR risk evaluated by weighted logistic regression

As displayed in Table 2, weighted logistic regression was applied to analyze the association between each metal and DR risk after adjusting for all covariates. When considering the concentrations of Co and Sb as continuous variables, an increase of one unit in Ln-Co and Ln-Sb concentrations resulted in a 48.9 and 47.5% increase in the risk of DR, respectively (all *p* < 0.05). In addition, a positive correlation was observed between Sb and DR when metal concentrations were divided into quartiles (*p* for trend = 0.036). Notably, there was a significantly positive correlation found between DR risk and Hg concentration in the third quartile (Q3) (OR = 2.322, 95% CI: 1.158–4.655, *p* = 0.018), whereas no significant correlation was detected for concentrations in the highest quartile (Q4).

**Table 2:** Associations of single urinary metals with DR risk in the study population.

Metal (μg/g creatinine)	Continuous		Q1	Q2		Q3		Q4		*p* for trend
	OR (95% CI)	*p* value	OR (95% CI)	OR (95% CI)	*p* value	OR (95% CI)	*p* value	OR (95% CI)	*p* value	
Ba										
Total	0.974 (0.809, 1.171)	0.773	ref	0.574 (0.336, 0.980)	0.042	0.836 (0.472, 1.482)	0.536	0.914 (0.504, 1.655)	0.763	0.868
Well-controlled	0.872 (0.671, 1.133)	0.301	ref	0.712 (0.326, 1.552)	0.387	**0.274 (0.120, 0.627)**	**0.003**	0.840 (0.349, 2.021)	0.693	0.537
Poorly controlled	1.083 (0.854, 1.374)	0.506	ref	**0.441 (0.215, 0.906)**	**0.027**	1.411 (0.671, 2.968)	0.359	1.007 (0.465, 2.178)	0.986	0.359
Co										
Total	**1.489 (1.064, 2.082)**	**0.021**	ref	0.917 (0.529, 1.589)	0.755	1.021 (0.602, 1.733)	0.937	1.547 (0.849, 2.819)	0.152	0.145
Well-controlled	1.508 (0.956, 2.377)	0.076	ref	0.717 (0.334, 1.539)	0.388	0.761 (0.359, 1.613)	0.472	1.798 (0.829, 3.901)	0.135	0.129
Poorly controlled	1.427 (0.940, 2.167)	0.094	ref	0.994 (0.464, 2.130)	0.988	1.190 (0.551, 2.569)	0.654	1.318 (0.503, 3.454)	0.569	0.494
Cs										
Total	1.294 (0.799, 2.096)	0.292	ref	0.696 (0.368, 1.316)	0.261	0.934 (0.502, 1.737)	0.827	1.281 (0.668, 2.458)	0.452	0.317
Well-controlled	0.790 (0.467, 1.336)	0.374	ref	0.616 (0.256, 1.483)	0.276	0.461 (0.184, 1.157)	0.098	0.802 (0.408, 1.576)	0.517	0.457
Poorly controlled	1.751 (0.948, 3.231)	0.073	ref	0.666 (0.269, 1.649)	0.374	1.722 (0.788, 3.765)	0.170	1.755 (0.694, 4.437)	0.231	0.106
Mo										
Total	0.947 (0.651, 1.376)	0.771	ref	1.015 (0.502, 2.053)	0.967	0.901 (0.439, 1.853)	0.775	1.073 (0.555, 2.075)	0.832	0.945
Well-controlled	1.505 (0.764, 2.965)	0.233		1.219 (0.386, 3.853)	0.732	1.828 (0.564, 5.923)	0.310	2.457 (0.802, 7.528)	0.114	0.092
Poorly controlled	0.630 (0.393, 1.009)	0.055	ref	0.870 (0.357, 2.120)	0.756	0.542 (0.188, 1.565)	0.253	0.562 (0.249, 1.272)	0.164	0.12
Sb										
Total	**1.475 (1.084, 2.008)**	**0.014**	ref	0.799 (0.426, 1.500)	0.481	1.471 (0.770, 2.809)	0.239	1.685 (0.948, 2.992)	0.075	**0.036**
Well-controlled	1.442 (0.961, 2.165)	0.077	ref	0.586 (0.258, 1.330)	0.198	1.566 (0.646, 3.798)	0.316	1.749 (0.747, 4.093)	0.194	0.094
Poorly controlled	**1.596 (1.022, 2.493)**	**0.040**	ref	0.837 (0.346, 2.026)	0.690	1.479 (0.617, 3.549)	0.375	1.632 (0.747, 3.561)	0.215	0.121
Tu										
Total	1.101 (0.807, 1.502)	0.538	ref	**0.485 (0.264, 0.891)**	**0.02**	1.169 (0.619, 2.210)	0.627	1.148 (0.598, 2.202)	0.675	0.299
Well-controlled	1.643 (0.999, 2.703)	0.051	ref	0.573 (0.260, 1.265)	0.165	**2.405 (1.075, 5.381)**	**0.033**	2.065 (0.802, 5.315)	0.131	**0.031**
Poorly controlled	0.850 (0.572, 1.262)	0.414	ref	0.474 (0.216, 1.041)	0.062	0.760 (0.303, 1.908)	0.554	0.832 (0.337, 2.052)	0.685	0.861
Tl										
Total	1.091 (0.737, 1.617)	0.442	ref	1.087 (0.621, 1.902)	0.768	0.849 (0.455, 1.583)	0.603	1.053 (0.619, 1.792)	0.848	0.838
Well-controlled	1.068 (0.605, 1.884)	0.818	ref	1.089 (0.558, 2.124)	0.801	0.921 (0.356, 2.380)	0.863	0.780 (0.371, 1.639)	0.507	0.461
Poorly controlled	1.050 (0.652, 1.690)	0.838	ref	1.155 (0.497, 2.688)	0.734	0.834 (0.361, 1.925)	0.666	1.280 (0.626, 2.617)	0.493	0.742
Pb										
Total	1.235 (0.914, 1.668)	0.167	ref	1.202 (0.658, 2.198)	0.545	1.236 (0.599, 2.551)	0.562	1.744 (0.954, 3.191)	0.07	0.116
Well-controlled	1.253 (0.827, 1.900)	0.283	ref	1.188 (0.463, 3.048)	0.717	1.614 (0.680, 3.831)	0.274	1.546 (0.631, 3.789)	0.336	0.219
Poorly controlled	1.229 (0.790, 1.910)	0.355	ref	1.249 (0.560, 2.786)	0.581	1.081 (0.429, 2.722)	0.868	2.099 (0.890, 4.951)	0.089	0.215
Cd										
Total	0.882 (0.639, 1.218)	0.441	ref	0.921 (0.454, 1.866)	0.816	1.046 (0.524, 2.091)	0.897	0.829 (0.423, 1.628)	0.583	0.714
Well-controlled	0.846 (0.543, 1.318)	0.455	ref	0.953 (0.351, 2.585)	0.924	0.540 (0.215, 1.351)	0.185	0.683 (0.279, 1.670)	0.398	0.237
Poorly controlled	0.896 (0.593, 1.353)	0.595	ref	0.951 (0.375, 2.411)	0.915	1.520 (0.625, 3.699)	0.351	0.949 (0.368, 2.446)	0.913	0.779
Hg										
Total	1.110 (0.909, 1.356)	0.302	ref	1.605 (0.794, 3.248)	0.190	**2.322 (1.158, 4.655)**	**0.018**	1.539 (0.727, 3.259)	0.256	0.13
Well-controlled	**1.509 (1.157, 1.967)**	**0.003**	ref	1.638 (0.596, 4.503)	0.334	**3.213 (1.377, 7.498)**	**0.008**	**3.608 (1.695, 7.681)**	**0.001**	**<0.001**
Poorly controlled	0.904 (0.694, 1.177)	0.447	ref	1.773 (0.709, 4.431)	0.216	2.329 (0.958, 5.658)	0.062	0.742 (0.263, 2.091)	0.567	0.731

Models were adjusted for age, sex, ethnicity, education, poverty income ratio, body mass index, drinking alcohol status, smoking status, glycemic control, hypertension and CKD. Continuous, Ln-transformed concentration of metal; CI, confidence interval; OR, odds ratio; Q, quartile; Ref, reference. *p* value was calculated by weighted logistic regression. Bold: *p* < 0.05.

Subgroup analysis based on the level of glycemic control was also performed (Table 2). In the poorly-controlled group, Sb exerted the most significant effect (OR = 1.596, 95% CI: 1.022–2.493, *p* = 0.04), but this relationship was not significantly different from that in the well-controlled group. In the well-controlled group, Ba concentration in Q3 (OR = 0.274, 95% CI: 0.120–0.627, *p* = 0.003) was significantly and negatively correlated with DR risk, but Ba concentration in Q4 did not. Both Hg concentrations in Q4 and Ln-Hg significantly increased the risk of DR (OR = 3.608, 95% CI: 1.695–7.681, *p* = 0.001; OR = 1.509, 95% CI: 1.157–1.967, *p* = 0.003) in the well-controlled group. Other metals have not been shown to have a meaningful association with DR.

To account for the potential influence of other heavy metals, weighted logistic regression models that considered all heavy metals were applied. As demonstrated in Supplementary Table 2, Hg concentration in Q3 significantly increased the risk of DR (OR = 2.407, 95% CI: 1.264–4.585, *p* = 0.008). Additionally, each per-unit increase in Ln-Co and Ln-Sb concentrations led to a 62.7 and 42.7% higher risk of DR, respectively (all *p* < 0.05).

### 3.4 Associations between heavy metal mixtures and DR risk evaluated by WQS regression

**Figure 2:**
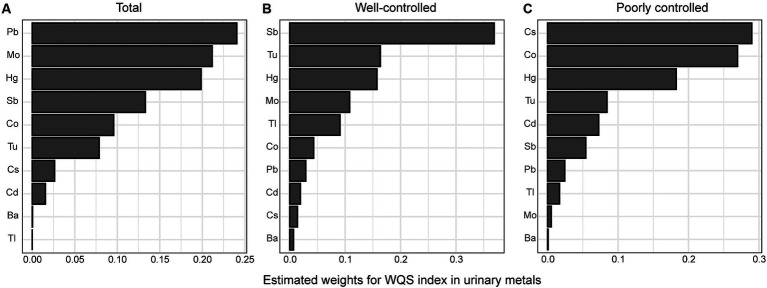
Estimated weights of heavy metals for DR by WQS models were adjusted for age, sex, ethnicity, education, poverty income ratio, body mass index, drinking alcohol status, smoking status, glycemic control, hypertension and CKD. **(A)** total population, **(B)** good glycemic control group, **(C)** poor glycemic control group. Ba, Barium; Cd, Cadmium; Co, Cobalt; Cs, Cesium; Mo, Molybdenum; Pb, lead; Sb, Antimony; Tl, Thallium; Tu, Tungsten; lead, Pb; mercury, Hg.

WQS regression was conducted to investigate the correlation between heavy metal mixtures and DR risk while adjusting for all covariates. In our study, the WQS index was positively correlated with DR risk (OR = 1.5, 95%CI: 1.07–2.10, *p* = 0.019). In the subgroup analysis stratified by glycemic control, the correlation between exposure to heavy metals and DR risk was not statistically significant in either the well-controlled or poorly-controlled group (all *p* > 0.05). Among the 10 heavy metals, Pb, Mo, Hg, Sb, and Co exhibited the highest weight in the whole population (Figure 2A). In the well-controlled group, Sb was determined to be the highest weighted metal (Figure 2B), whereas Cs and Co were the most heavily weighted metals in the poorly-controlled group (Figure 2C).

### 3.5 Associations between heavy metal mixtures and DR risk evaluated by the BKMR model

**Figure 3:**
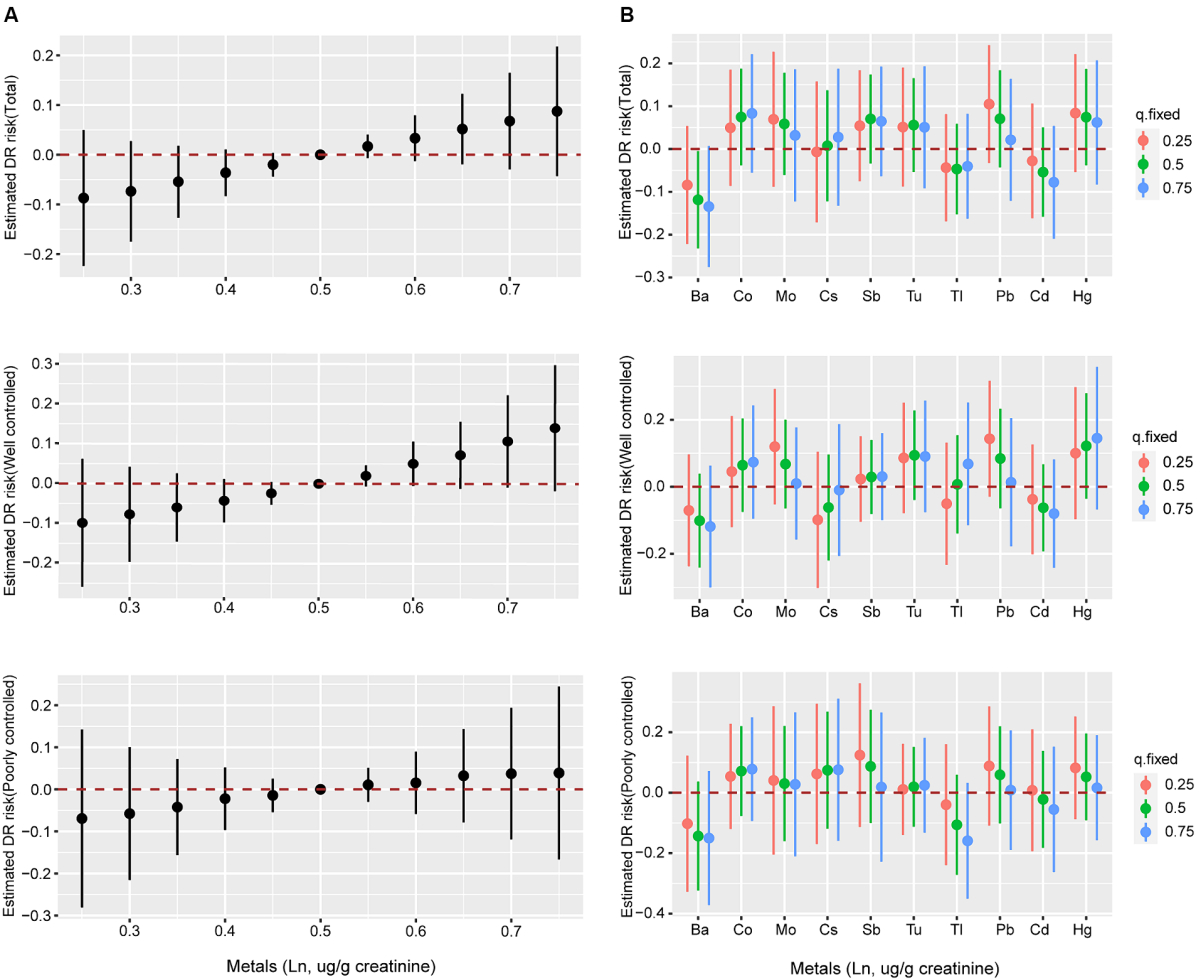
The associations of metal mixtures and DR risk evaluated by BKMR model. **(A)** The joint effects of heavy metal mixtures on DR risk were estimated by BKMR models in total population and subgroups, when all the metals at particular percentiles were compared to all the metals at their 50th percentile. **(B)** Associations of single heavy metals with DR risk were estimated by BKMR models in total population and subgroups, when other all metals were held at their corresponding 25th (red), 50th (green) or 75th (blue) percentile, respectively. Models were adjusted for age, sex, ethnicity, education, poverty income ratio, body mass index, drinking alcohol status, smoking status, glycemic control, hypertension and CKD. Ba, Barium; Cd, Cadmium; Co, Cobalt; Cs, Cesium; Mo, Molybdenum; Pb, lead; Sb, Antimony; Tl, Thallium; Tu, Tungsten; lead, Pb; mercury, Hg.

Although no statistically significant effect was observed, there was a discernible increase in the risk of DR when heavy metal mixture concentrations were at or exceeded the 60th percentile (Figure 3A). Similar associations were observed in both the well-controlled and poorly-controlled groups, as depicted in Figure 3A. When concentrations of other metals were fixed at the 25th, 50th, and 75th percentiles, Co, Mo, Sb, Tu, Pb, and Hg concentrations all displayed a positive correlation with DR risk, with PIP values exceeding 0.55 (Figure 3B and Supplementary Table 3). Similar trends were observed in both the well-controlled and poorly-controlled groups, although the correlations were not statistically significant, as delineated in Figure 3B. The univariate exposure-response relationship exhibited a monotonic upward trend between DR and Co, Sb, Tu, and Pb concentrations when the other metals were fixed at the median level. However, Ba, Tl, and Cd displayed a monotonic downward trend (Supplementary Figure 2). Based on the moderate correlations between some metals, the interactions among the 10 heavy metals were separately analyzed, revealing underlying interactions between specific heavy metals. Supplementary Figure 3 delineates that Co interacts with Cs, Sb, Tl, Hg, and Pb, whilst Sb interacts with Tu, Cd, and Hg, and Hg interacts with most metals.

### 3.6 Associations between concentrations of heavy metals and DR risk in the RCS analysis

**Figure 4:**
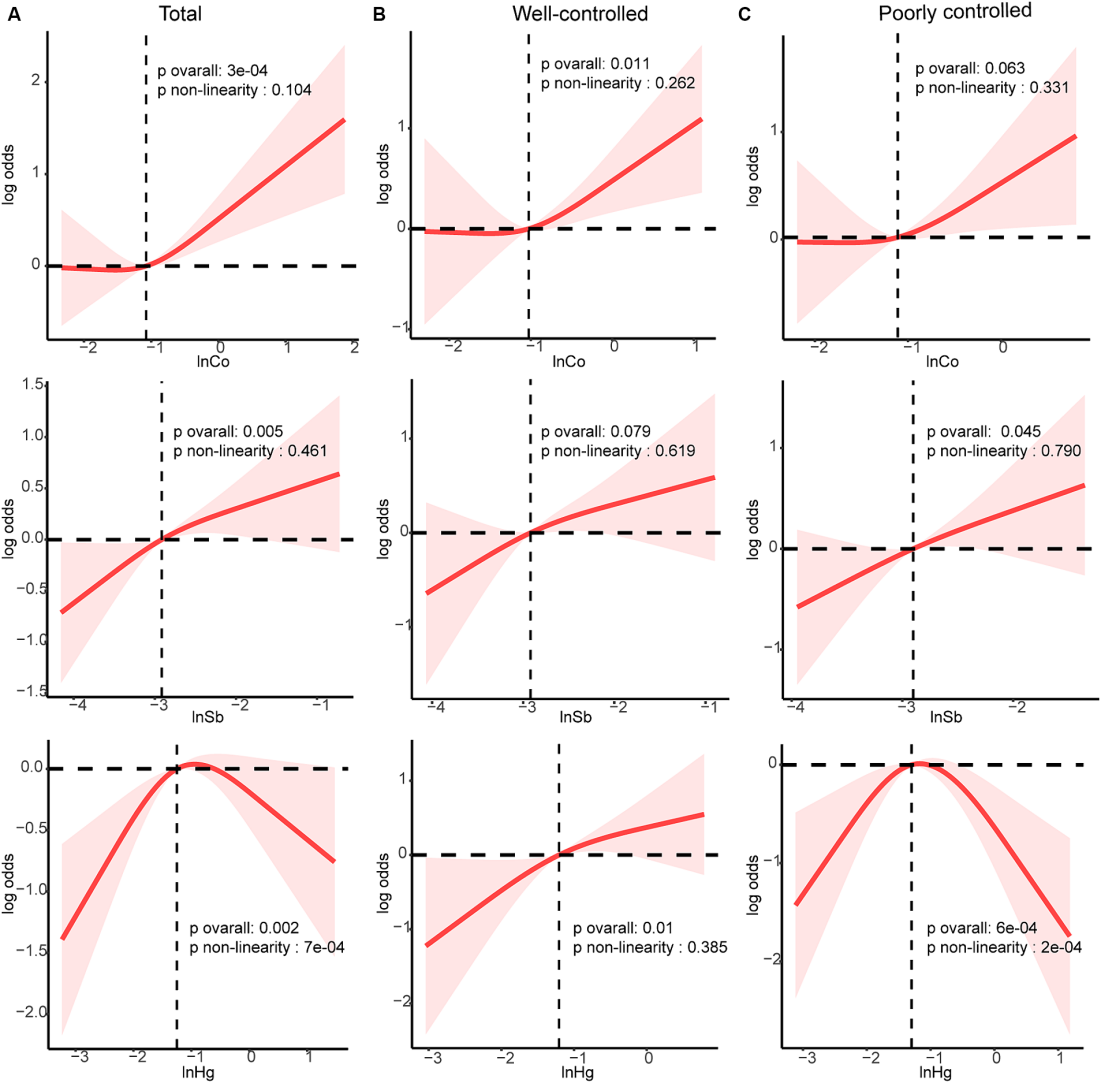
Dose–response relationship between Sb, Co and Hg with DR risk were estimated by RCS models in total population and subgroups. Models were adjusted for age, sex, ethnicity, education, poverty income ratio, body mass index, drinking alcohol status, smoking status, glycemic control, hypertension and CKD. **(A)** total population, **(B)** good glycemic control group, **(C)** poor glycemic control group. Solid line, odds ratios; red-shade, 95% CI.

Co, Sb, and Hg concentrations, which were closely related to DR risk, were further analyzed. The dose–response relationships were evaluated in the RCS analysis (Figure 4). Linear and positive associations with DR risk were identified for the Ln-transformed concentrations of Co and Sb (all *p*_nonlinearity_ > 0.05, all *p*_overall_ < 0.05), except for Hg (*p*_nonlinearity_ = 7e-04). In both the well-controlled and poorly-controlled groups, Co and Sb concentrations had a linear relationship with DR risk. In the well-controlled group, the risk of DR generally increased with increasing Co concentration (*p*_overall_ = 0.011, *p*_nonlinearity_ = 0.262). Conversely, in the poorly-controlled group, a positive linear dose correlation between Sb concentrations and DR risk was noted (*p*_overall_ = 0.045, *p*_nonlinearity_ = 0.790). Finally, a linear and positive correlation between Hg concentrations and the risk of DR was observed solely in the well-controlled group (*p*_overall_ = 0.01, *p*_nonlinearity_ = 0.385).

## 4 Discussion

To the best of our knowledge, this is the first cross-sectional study to investigate the effects of urinary heavy metals on the risk of DR in a substantial, nationally representative sample utilizing various statistical techniques. Herein, the results of weighted logistic regression demonstrated that Co, Sb, and Hg were associated with DR risk in the single-metal model, and this association was also observed in the multi-metal model. Both the WQS and BMKR models suggested that mixed exposure to these 10 heavy metals was positively associated with DR risk. Furthermore, the results of the RCS regression displayed a linear and positive correlation between Co and Sb and DR risk but a non-linear correlation between Hg concentrations and DR risk. The results of the subgroup analyses signaled that the aforementioned associations appeared to be more pronounced in the poorly-controlled group.

Co is widely distributed in nature. Humans are commonly exposed to Co through multiple routes, including food, environmental pollution, occupational exposure, and medical interventions (34). Besides, it is an essential element for human health, serving as the metallic component of vitamin B12 (35). Despite its vital importance, its potential toxicity can elicit adverse health effects after prolonged exposure. A cross-sectional study identified a positive correlation between diabetes and urinary Co concentrations (16). Consistently, a study discovered a strong correlation between elevated urinary Co levels and increased levels of FPG and HbA1c in male participants (36). At the same time, Cancarini et al. concluded that the Co concentration in the tear film of diabetic patients was higher than that in the control group (37). This increase may be attributed to the rise in conjunctival vascular permeability caused by diabetes, similar to the increase in retinal vascular permeability driven by diabetes (a characteristic of DR) (38). In our study, diabetic patients with higher urinary concentrations of Co were more likely to develop DR. This may be ascribed to the oxidizing effect of Co promoting the formation of free radicals, inducing oxidative stress responses, and contributing to mitochondrial dysfunction (39). Of note, accumulating evidence suggests that oxidative damage and mitochondrial dysfunction promote the development of DR (40, 41).

Sb is a toxic heavy metal to which humans are primarily exposed through the consumption of food and air, soil, and water exposure. Numerous studies have demonstrated that it exerts various toxic effects on vital organs, including but not limited to the pancreas, liver, lungs, intestines, and spleen (42). A cross-sectional study conducted in the USA demonstrated an association between urine Sb concentrations and insulin resistance (16). Likewise, a cross-sectional study conducted in China found that urinary Sb levels are linked to an increased risk of increased FPG levels, impaired fasting glucose, and diabetes (18). Furthermore, a prospective study indicated that pregnant women with higher exposure to Sb may face an increased risk of gestational diabetes mellitus (43). Xiao et al. reported that elevated urinary Sb concentrations are linked to a higher incidence of type 2 diabetes, and this process is partially implicated in oxidative DNA damage (44). These studies collectively imply that Sb exposure may contribute to the development of diabetes. However, to date, there has been no report on the correlation between Sb levels and DR. Our study uncovered that diabetic patients with elevated urinary Sb levels have a significantly increased risk of developing DR, especially in those with poor glycemic control.

Hg is a highly toxic heavy metal that can cause significant harm to numerous organs in the human body (45). Currently, research on the relationship between Hg levels and diabetes risk remains inconclusive. Earlier studies found no significant association between blood or urine Hg concentrations and an increased risk of diabetes in adults (46–48). However, Tsai et al. observed a significant increase in Hg levels in the red blood cells of type 2 diabetes patients compared to those without the condition (49). A large prospective cohort study determined that people with high Hg exposure during early adulthood were at a higher risk of developing diabetes in the future (50). Furthermore, research has demonstrated that Hg can selectively affect *β* cells in the pancreas, resulting in cellular dysfunction and apoptosis (51). In this study, urine Hg levels among diabetes patients with DR were higher compared to those without DR, although the difference was not statistically significant. Additionally, a clear non-monotonic relationship was identified between Hg levels and the risk of DR. This may be due to the fact that the chief source of human exposure to Hg is the consumption of marine fish, which are rich in omega-3 fatty acids that counteract the toxicity of Hg (52, 53). A significant positive correlation was observed between Hg concentrations and DR risk only in the well-controlled group, warranting further investigation.

Heavy metals stimulate reactive oxygen species production, leading to oxidative damage, which is one of the mechanisms involved in disease development (54). The retina is a high-oxygen-consuming tissue that is highly susceptible to damage from oxidative stress. Previous studies have shown a robust correlation between oxidative stress and retinal vascular impairment under hyperglycemic conditions (55). However, the role of heavy metals in DR development via oxidative stress mechanisms remains unclear. Thus, further experimental validation is necessary.

This study has several advantages. Firstly, it is the first study that investigated the correlation between urinary heavy metals and DR risk, considering both the single and co-exposure effects of heavy metals. In contrast, Zhang et al. focused on the relationship between blood heavy metals and DR risk without exploring the combined effects of heavy metals on DR (15). Furthermore, our study included a higher number of metals than those conducted by Zhang et al. (15) and reported for the first time that urinary levels of Co, Sb, and Hg may be associated with DR risk. Secondly, weighted logistic regression, WQS regression model, BMKR model, and RCS regression were employed to investigate the correlation between heavy metals and DR risk in a diabetes population from multiple perspectives. These statistical methods have been extensively utilized to explore the effects of heavy metals on diabetes and hypertension (21, 56). Finally, previous research has demonstrated a correlation between heavy metal exposure and HbA1c levels. High HbA1c level has been established as a risk factor for DR. Therefore, subgroup analysis was initially conducted based on glycemic control (determined by HbA1c value) to investigate the correlation between urine levels of heavy metals and DR risk.

Nevertheless, some limitations of this study merit acknowledgment. Given the inherent shortcomings of cross-sectional studies (57), this study could not establish a causal relationship between metal exposure and the risk of DR. Furthermore, relying on self-report questionnaires for DR diagnosis may introduce recall bias. In addition, the dataset lacked precise information regarding retinopathy severity, thereby limiting further analysis. The concentrations of heavy metals in urine are affected by various factors, not all of which were accounted for in this study, potentially compromising the reliability of the results. Additionally, selection bias selection bias may be present due to missing data and the exclusion of participants with incomplete information. Therefore, further studies are necessitated to corroborate our findings and to investigate the relationship between metal concentrations and DR severity, as well as to elucidate the underlying mechanisms by which metals affect DR.

## 5 Conclusion

Overall, our cross-sectional study demonstrated that several heavy metals, including Co, Sb, and Hg, were significantly associated with an elevated risk of DR. Furthermore, a linear and positive correlation was observed between the concentrations of Co and Sb and the risk of DR, while a non-linear correlation was identified between Hg levels and DR risk. The results of the subgroup analyses signaled that the aforementioned associations appeared to be more pronounced in the poorly-controlled group. The results of the mixture exposure analysis indicated a positive association between mixed metal exposure and the risk of DR. This association was observed in both the well-controlled group and the poorly-controlled group. Due to the limitations of the present study, subsequent investigations are required to substantiate these findings and to clarify the mechanisms by which heavy metals affect DR.

## Data availability statement

The original contributions presented in the study are included in the article/Supplementary material, further inquiries can be directed to the corresponding author.

## Author contributions

CM: Conceptualization, Formal analysis, Methodology, Writing – original draft, Writing – review & editing. CG: Data curation, Formal analysis, Methodology, Writing – original draft, Writing – review & editing. CC: Formal analysis, Methodology, Writing – review & editing. SH: Formal analysis, Methodology, Writing – original draft. DL: Data curation, Methodology, Writing – original draft. QQ: Conceptualization, Supervision, Writing – review & editing.
