# Supplementary material for: Associations of heavy metal exposure with diabetic retinopathy in the U.S. diabetic population: a cross-sectional study
Source: Front Public Health. 2024 Aug 1;12:1401034. doi: 10.3389/fpubh.2024.1401034 (PMC11324498; doi:10.3389/fpubh.2024.1401034)
Supplement: Supplementary file 1 [file Data_Sheet_1.docx]

Supplementary Material

Associations of heavy metal exposure with incident diabetic retinopathy in the U.S. population with diabetes: A cross-sectional study

Chunren Meng^1#,^ Chufeng Gu^1#^, Shuai He^1^, Chunyang Cai^1^, Dongwei Lai^1^, Qinghua Qiu^1-3*^

^1^Department of Ophthalmology, Shanghai General Hospital, Shanghai Jiao Tong University School of Medicine, National Clinical Research Center for Eye Diseases; Shanghai Clinical Research Center for Eye Diseases; Shanghai Key Laboratory of Ocular Fundus Diseases; Shanghai Engineering Center for Visual Science and Photomedicine; Shanghai engineering center for precise diagnosis and treatment of eye diseases, Shanghai, PR China

^2^Department of Ophthalmology, Tong Ren Hospital, Shanghai Jiao Tong University School of Medicine, Shanghai, PR China

^3^Department of Ophthalmology, Shigatse People’s Hospital, Shigatse, Xizang, PR China

*** Correspondence:** Qinghua Qiu: qinghuaqiu@163.com

**Supplementary Table 1**. Distributions of urine metals in the study population.

|  |  | Total (N=1146) | | | |  | Non-DR(N=907) | | | |  | DR(N=239) | | | |  |
| --- | --- | --- | --- | --- | --- | --- | --- | --- | --- | --- | --- | --- | --- | --- | --- | --- |
| metals  (μg/g creatinine) | Detection  rates (%) | Median (P25,P75) | Min | Mean | Max |  | Median (P25,P75) | Min | Mean | Max |  | Median (P25,P75) | Min | Mean | Max | *p* value |
| Ba | 95.63 | 1.10(0.54,2.02) | 0.02 | 1.64 | 97.77 |  | 1.10(0.55,2.03) | 0.02 | 1.83 | 97.77 |  | 1.14(0.43,1.95) | 0.03 | 1.40 | 9.69 | 0.603 |
| Co | 95.63 | 0.38(0.24,0.54) | 0.03 | 0.64 | 62.34 |  | 0.38(0.25,0.54) | 0.03 | 0.55 | 34.76 |  | 0.39(0.24,0.58) | 0.08 | 0.96 | 62.34 | 0.54 |
| Cs | 95.63 | 4.73(3.38,6.44) | 0.64 | 5.17 | 111.45 |  | 4.71(3.42,6.36) | 0.64 | 5.19 | 111.45 |  | 0.39(0.24,0.5) | 0.73 | 5.08 | 27.43 | 0.681 |
| Mo | 95.63 | 42.54(27.97,60.46) | 2.71 | 52.38 | 494.12 |  | 42.63(27.99,60.00) | 2.71 | 52.29 | 494.12 |  | 42.22(27.59,61.75) | 7.20 | 52.75 | 243.59 | 0.931 |
| Sb | 95.63 | 0.05(0.04,0.08) | 0.01 | 0.08 | 1.15 |  | 0.05(0.04,0.08) | 0.01 | 0.07 | 1.15 |  | 0.06(0.04,0.09) | 0.01 | 0.08 | 0.84 | **0.013** |
| Tu | 95.56 | 0.08(0.04,0.13) | 0.01 | 0.11 | 4.71 |  | 0.07(0.04,0.12) | 0.01 | 0.11 | 4.71 |  | 0.09(0.04,0.14) | 0.01 | 0.12 | 2.15 | 0.394 |
| Tl | 95.63 | 0.15(0.11,0.23) | 0.02 | 0.18 | 1.64 |  | 0.15(0.11,0.23) | 0.02 | 0.18 | 1.19 |  | 0.14(0.10,0.23) | 0.02 | 0.17 | 1.64 | 0.584 |
| Pb | 93.61 | 0.41(0.25,0.63) | 0.06 | 0.61 | 10.14 |  | 0.41(0.25,0.63) | 0.06 | 0.61 | 7.70 |  | 0.42(0.23,0.65) | 0.07 | 0.64 | 10.14 | 0.899 |
| Cd | 95.63 | 0.25(0.16,0.44) | 0.02 | 0.41 | 5.49 |  | 0.25(0.16,0.44) | 0.02 | 0.41 | 5.49 |  | 0.25(0.14,0.43) | 0.03 | 0.39 | 1.76 | 0.527 |
| Hg | 95.9 | 0.31(0.16,0.58) | 0.02 | 0.56 | 24.00 |  | 0.29(0.15,0.60) | 0.02 | 0.56 | 24.00 |  | 0.33(0.18,0.56) | 0.05 | 0.54 | 12.57 | 0.303 |

N, numbers of subject，DR, diabetic retinopathy; Ba, Barium; Co, Cobalt; Cs, Cesium; Mo, Molybdenum; Sb, Antimony; Tu, Tungsten; Tl, Thallium; Pb, lead; Cd, Cadmium; **Bold**: *p* < 0.05.

**Supplementary Table 2.** Associations of multiple urinary metals with DR risk after adjustment for other metals in the study population.

| Metals  **(μg/g creatinine)** | Q1 |  | Q2 | |  | Q3 | |  | Q4 | |  | Continuous | |
| --- | --- | --- | --- | --- | --- | --- | --- | --- | --- | --- | --- | --- | --- |
|  | OR (95% CI) |  | OR (95% CI) | *p* value |  | OR (95% CI) | *p* value |  | OR (95% CI) | *p* value |  | OR (95% CI) | *p* value |
| Ba | Ref |  | **0.527**  **(0.302,0.920)** | **0.025** |  | 0.651  (0.349,1.216) | 0.175 |  | 0.585  (0.283,1.211) | 0.146 |  | 0.783  (0.602,1.017) | 0.066 |
| Co | Ref |  | 1.170  (0.675,2.027) | 0.57 |  | 1.163  (0.667,2.028) | 0.59 |  | 1.702  (0.846,3.422) | 0.133 |  | **1.627**  **(1.111,2.384)** | **0.013** |
| Cs | Ref |  | 0.727  (0.385,1.375) | 0.321 |  | 0.884  (0.468,1.670) | 0.699 |  | 1.259  (0.609,2.606) | 0.528 |  | 1.381  (0.742,2.573) | 0.305 |
| Mo | Ref |  | 1.166  (0.584,2.328) | 0.658 |  | 0.903  (0.456,1.789) | 0.766 |  | 1.001  (0.520,1.928) | 0.998 |  | 0.840  (0.577,1.225) | 0.361 |
| Sb | Ref |  | 0.828  (0.416,1.649) | 0.587 |  | 1.358  (0.682,2.706) | 0.378 |  | 1.502(0.744,3.035) | 0.252 |  | **1.427**  **(1.037,1.964)** | **0.03** |
| Tu | Ref |  | **0.421**  **(0.221,0.805)** | **0.01** |  | 0.983  (0.523,1.848) | 0.957 |  | 0.851  (0.448,1.619) | 0.618 |  | 1.035  (0.751,1.427) | 0.831 |
| Tl | Ref |  | 0.966  (0.522,1.789) | 0.912 |  | 0.757  (0.375,1.528) | 0.431 |  | 0.787  (0.391,1.582) | 0.495 |  | 0.918  (0.579,1.455) | 0.713 |
| Pb | Ref |  | 1.149  (0.638,2.071) | 0.638 |  | 1.081  (0.549,2.130) | 0.819 |  | 1.413  (0.727,2.748) | 0.302 |  | 1.087  (0.771,1.532) | 0.63 |
| Cd | Ref |  | 0.900  (0.457,1.771) | 0.756 |  | 0.909  (0.451,1.832) | 0.787 |  | 0.705  (0.369,1.349) | 0.286 |  | 0.804  (0.581,1.112) | 0.184 |
| Hg | Ref |  | 1.635  (0.818,3.265) | 0.161 |  | **2.407**  **(1.264,4.585)** | **0.008** |  | 1.651  (0.773,3.526) | 0.192 |  | 1.124  (0.903,1.399) | 0.293 |

Models were adjusted for age, sex, ethnicity, education, Poverty Income Ratio, body mass index, drinking alcohol status, smoking status, glycemic control, hypertension and CKD. Continuous, Ln-transformed concentration of metal; CI: confidence interval; OR: odds ratio; Q, quartile; Ref, reference. P value was calculated by weighted logistic regression. **Bold: p < 0.05.**

**Supplementary Table 3.** The posteriori inclusion probability of single urinary metals in the study population.

|  | PIP value | | |
| --- | --- | --- | --- |
| Metals | Total | Well-controlled | Poorly controlled |
| (μg/g creatinine) |  |  |  |
| Ba | 0.6768 | 0.3752 | 0.37792 |
| **Co** | **0.6326** | 0.399 | 0.36528 |
| Mo | 0.5584 | 0.4442 | 0.24424 |
| Cs | 0.5128 | 0.4388 | 0.24136 |
| **Sb** | **0.649** | 0.2284 | **0.41688** |
| Tu | 0.6342 | 0.5462 | 0.2408 |
| Tl | 0.571 | 0.5206 | 0.34472 |
| Pb | 0.5814 | 0.4656 | 0.2356 |
| Cd | 0.5766 | 0.3528 | 0.29848 |
| Hg | 0.6018 | 0.558 | 0.27344 |

PIP, posteriori inclusion probability.


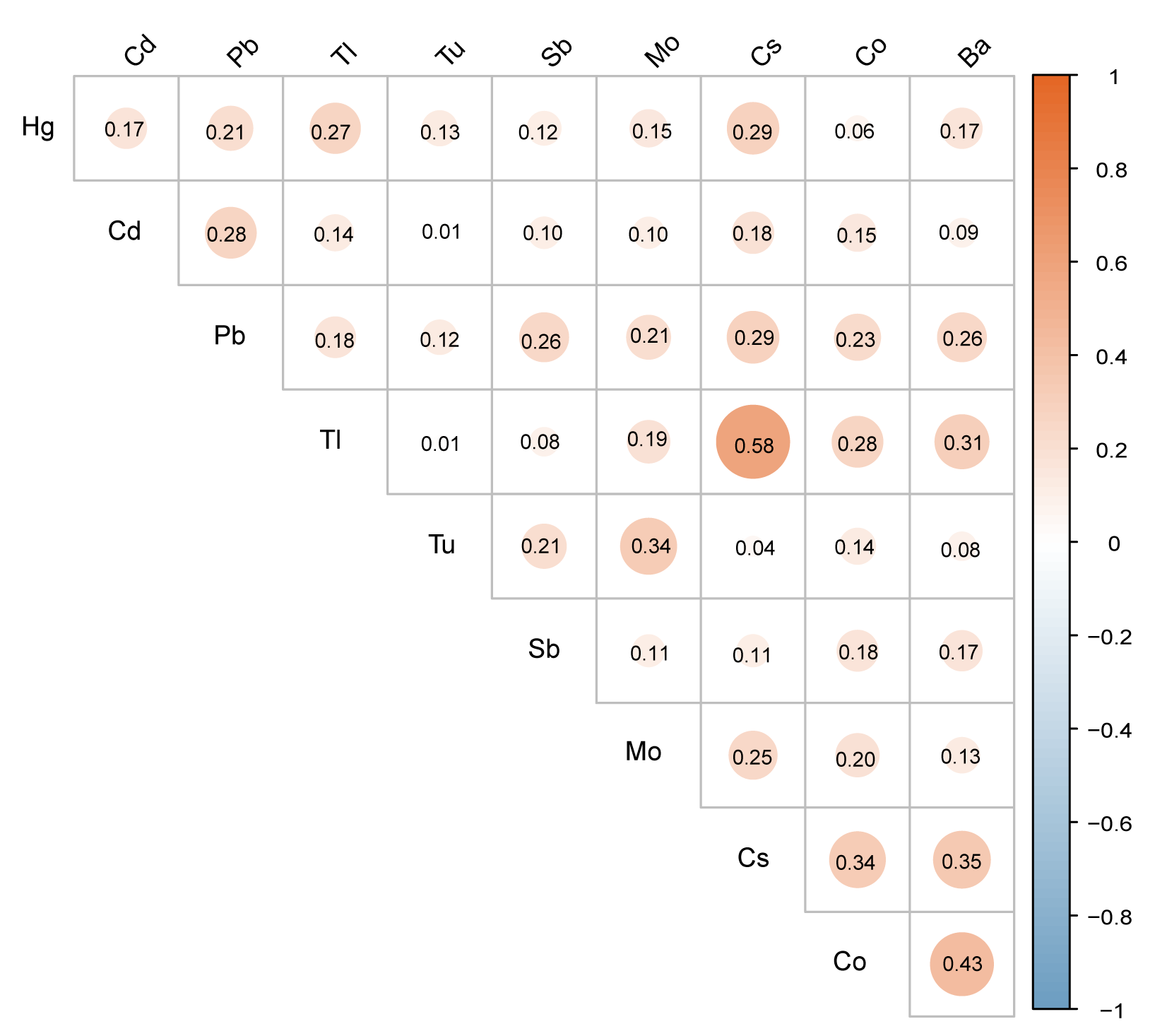


**Supplementary Figure 1.** Pearson’s correlation matrix among Ln-transformed heavy metals in the study population. Ba, Barium; Cd, Cadmium; Co, Cobalt; Cs, Cesium; Mo, Molybdenum; Pb, lead; Sb, Antimony; Tl, Thallium; Tu, Tungsten; lead, Pb; mercury, Hg.


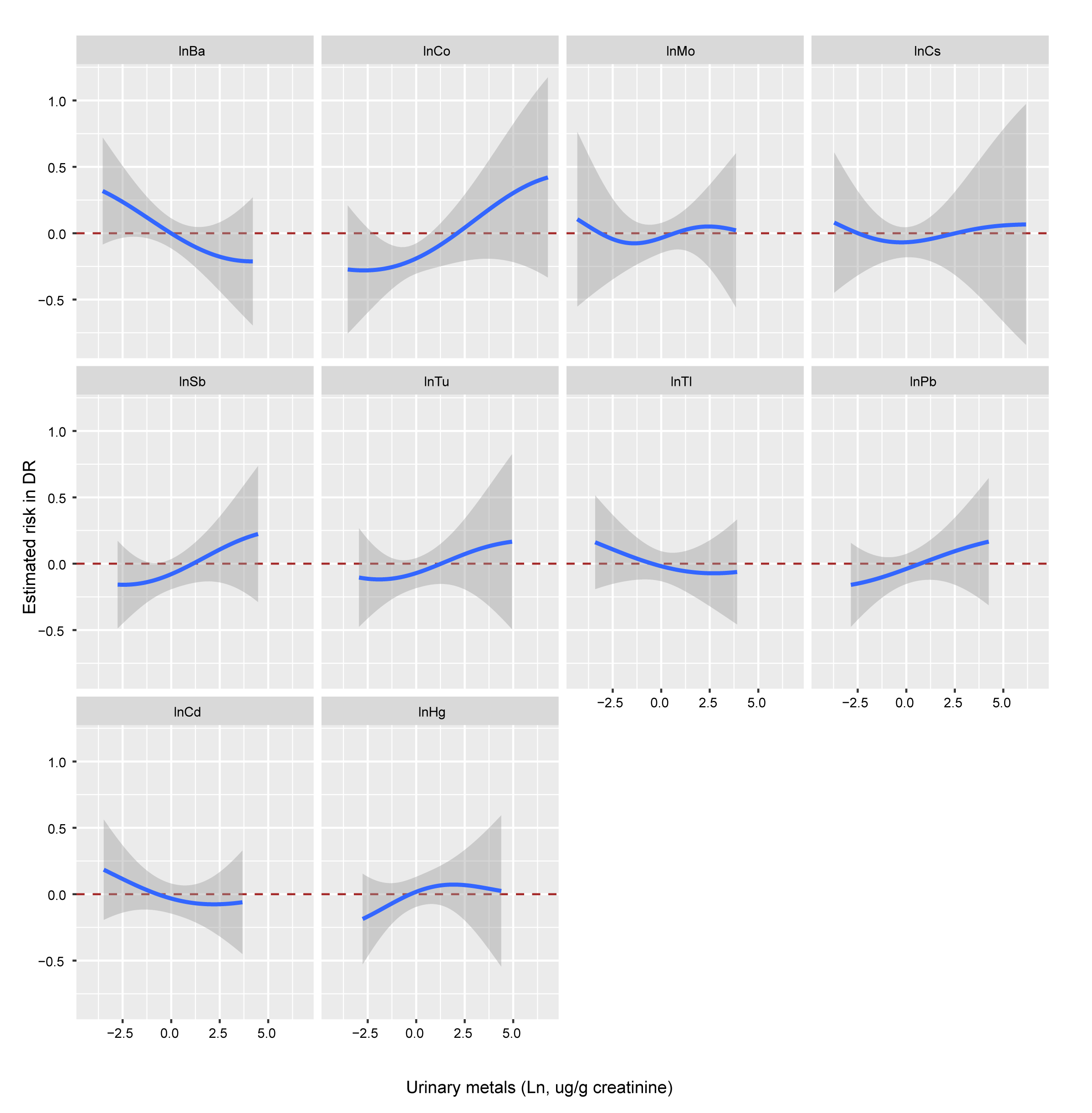


**Supplementary Figure 2**. Exposure-response relationship of heavy metal and DR based on BKMR .The univariate exposure-response relationship between individual heavy metal and DR based on Bayesian Kernel Machine Regression. Models were adjusted for age, sex, ethnicity, education, Poverty Income Ratio, body mass index, drinking alcohol status, smoking status, glycemic control, hypertension and CKD. Ba, Barium; Cd, Cadmium; Co, Cobalt; Cs, Cesium; Mo, Molybdenum; Pb, lead; Sb, Antimony; Tl, Thallium; Tu, Tungsten; lead, Pb; mercury, Hg.


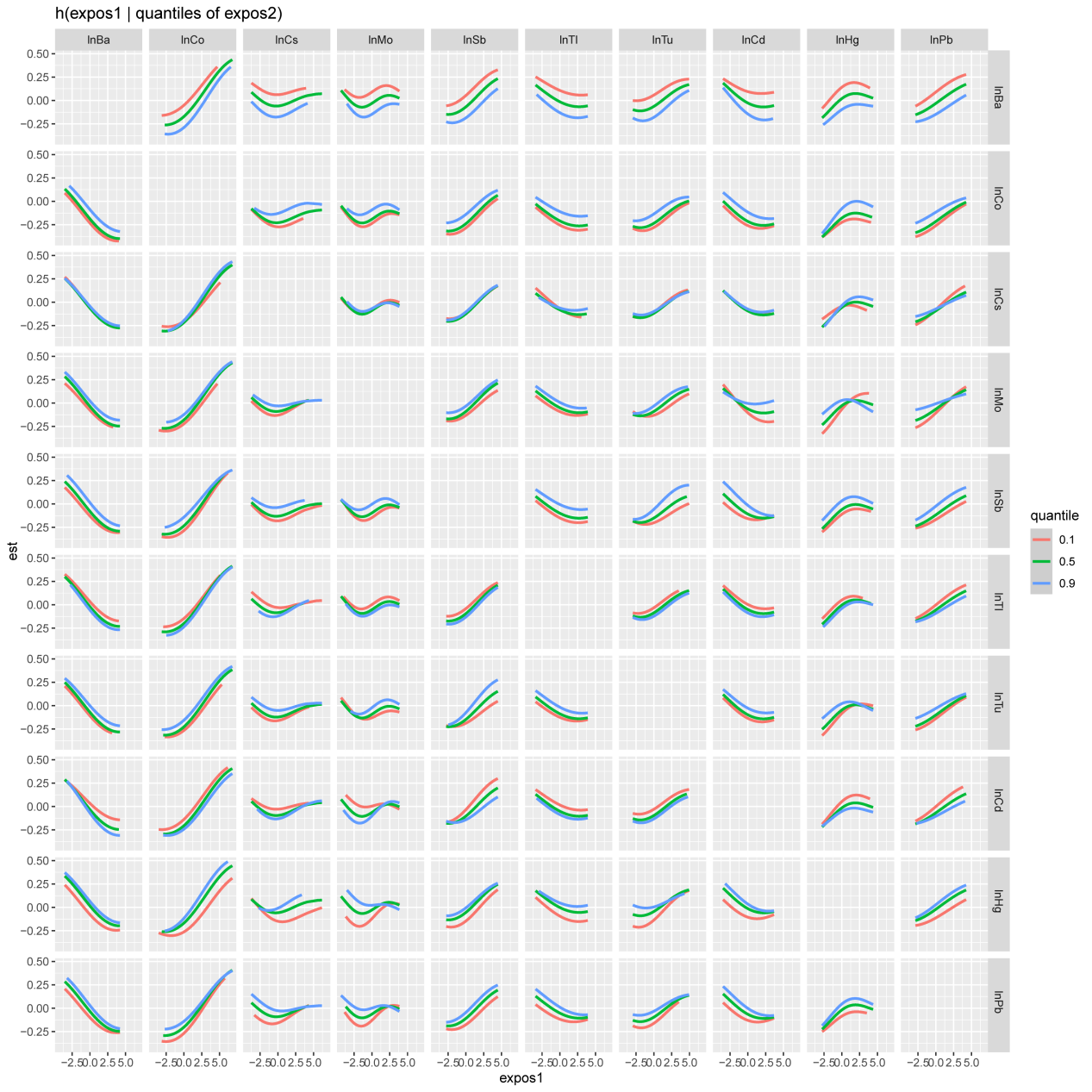


**Supplementary Figure 3.** The interaction of heavy metals for DR by BKMR models adjusted for age, sex, ethnicity, education, Poverty Income Ratio, body mass index, drinking alcohol status, smoking status, glycemic control, hypertension and CKD.
